# A Different Position: Protest Medicine and the Limits of Professional Neutrality

**DOI:** 10.1111/1467-9566.70243

**Published:** 2026-07-17

**Authors:** Cristian Montenegro

**Affiliations:** ^1^ Department of Global Health and Social Medicine King's College London London UK

## Abstract

Protests are a persistent feature of contemporary urban life, often producing mass injuries through police repression. Yet the role of medical care in these contexts—especially when provided by informal collectives—remains understudied. Drawing on 21 in‐depth interviews with members of volunteer medical brigades during Chile's 2019–2020 Estallido Social, this paper examines how care was improvised, coordinated and transformed under conditions of sustained police violence, where more than 3500 people were wounded and 36 died. The analysis explores how brigade members interpreted harm, navigated the collapse of medical neutrality, which was first denied by the state and then renounced by brigade members, and embedded rudimentary clinical practice within the protest itself. The paper proposes the concept of protest medicine. Unlike security‐based approaches that presuppose the legitimacy of state action, protest medicine is a grassroots, politically entangled form of medical care shaped by the spatial, ethical and affective logics of protest. In doing medicine outside and against the state, protest medicine foregrounds a form of healthcare rooted in proximity, solidarity and resistance rather than neutrality, regulation and order, opening space to rethink the social and political functions of medicine when the state is a source of harm.

## 1 Introduction

I arrived on the first day, alone, as a protester. That day was a massacre—I’m talking about October 18 and 19, those first days, what we witnessed was a massacre. So, on the second day, I came to the square with a backpack full of dressings, saline solution and a white helmet with a red cross made of tape. I went with a friend, just the two of us. Up to that point, no one was talking about brigades—there were no brigades yet. The only thing you could see were individuals offering care on the spot. That’s how I arrived. And although I came initially as a protester, when I saw the massacre unfolding before us, I saw two options: either go to the front‐line and fight the cops or take up *a different position*.

—Andrés Molina, chemist and rescue worker

Andrés’ decision to take up a ‘different position’ during the early days of Chile's 2019 uprising—to provide care, rather than engage in front‐line protest—offers a rationale for an ethic of political engagement in the face of suffering. That ‘different position’ also captures the role that health brigades came to occupy during the Estallido Social in relation to both medicine and the state. This paper explores the ways in which health brigades—a common phenomenon in protests worldwide—trouble conventional accounts of healthcare professionalism, opening a space for rethinking care outside and against the state.

Drawing on qualitative interviews with participants involved in medical brigades during Chile's 2019–2020 Estallido Social and building on literature from similar events in Egypt and Türkiye, this paper delineates protest medicine as a form of care that emerges from within collective action, shaped by the repression that calls it into being and by the solidarities enacted through it. Protest medicine, I argue, is an under‐theorised form of care in contemporary urban settings and a practice that forces a revisiting of classic sociological premises around the contours of medical professionalism, neutrality and the state.

The events that Andrés describes were part of a broader nationwide uprising known as the *Estallido Social*, triggered in October 2019 by a 4% rise in public transport fares (by 30 Chilean pesos). Initially led by secondary school students evading fares, the protests rapidly expanded into a multisited revolt against systemic inequality, institutional corruption and the enduring legacies of Chile's neoliberal political and economic model (Aranguez Muñoz and Sanhueza Huenupi 2021; Jerrems et al. 2022; Morales Quiroga 2020). The uprising was geographically dispersed and tactically diverse, with the Plaza Italia area (renamed Plaza Dignidad by protesters) as the epicentre (Jerrems et al. 2022; Santibáñez‐Rodríguez 2022). Although some actions were peaceful, others involved barricades, police confrontations and street appropriation through art and performance (Jerrems et al. 2022). The protests prompted a political agreement to pursue a new constitution, marking a turning point in Chile's democratic trajectory, even though later attempts to replace the dictatorship‐era text failed (Aranguez Muñoz and Sanhueza Huenupi 2021).

A defining element of the Estallido was the repressive response. Between October 2019 and March 2020, police, military and anti‐riot forces deployed so‐called non‐lethal ammunition, injuring more than 3700 people, including 2122 with gunshot wounds and 411 with ocular trauma; 34 people lost their vision and 36 were killed (Amnesty International 2020). More than 10,000 complaints of institutional violence were filed (Amnistía Internacional 2023). The violence, impunity and mistrust of institutions ‐including health‐spurred the formation of health brigades: multiple groups of 4–8 students and professionals with varying levels of medical or emergency training, providing on‐site physical and psychological care to protesters. These groups emerged spontaneously, refusing to trust institutions perceived as complicit. Many began with basic first aid but soon evolved into decentralized, collaborative care practices. As Andrés’ testimony suggests, many did not arrive as medics, but became medics *in* and *through* protest—occupying a ‘different position’ rooted in care rather than confrontation, yet inseparable from the collective act of resistance.

The Estallido reflected decades of simmering discontent. The slogan *“No son 30 pesos, son 30 años”* (It's not 30 pesos, it's 30 years) encapsulated a rejection of the neoliberal model imposed during the dictatorship, especially its marketised healthcare, education and pension systems (Aranguez Muñoz and Sanhueza Huenupi 2021). Prior student movements, environmental protests, and feminist organising laid the groundwork for mass mobilisation (Santibáñez‐Rodríguez 2022). The uprising also reflected broader crises of trust and institutional legitimacy that included corruption scandals (Morales Quiroga 2020).

### 1.1 Medical Rationalities of Protest: Risk, Disorder and Control

To understand the role of medical brigades in protests, two interlocked questions are relevant: What is protest from a medical and public health perspective? And how has medical care been framed in the context of public order concerns? Over recent decades, state‐based approaches to the health management of large gatherings—including protest—have exemplified a logic of protest‐as‐threat. Here, I examine two illustrative expressions: Mass Gathering Medicine (MGM) and Public Order Medicine (POM).

Mass Gathering Medicine (MGM), as defined by the World Health Organization, refers to medical planning for large groups gathered at specific locations for defined purposes over a limited time (Garbin et al. 2024; World Health Organization 2015). This includes sports events, music festivals, religious pilgrimages and other situations that, due to their massive scale, can strain existing health infrastructure (Yassine et al. 2022). Public Order Medicine (POM), on the other hand, focuses on providing medical care during violent urban disorders, understood as high‐threat environments “too dangerous for conventional medical assets and therefore necessitating specialist training, equipment and experience, distinct from those employed in a ballistic or explosive threat environment” (Brayford‐Harris et al. 2023, 17). This includes features specific to protest, such as dense crowds, direct physical assaults and projectiles.

MGM and POM are indifferent to causes or context, approaching protest as disruption and risk rather than as political action with historical context. This reflects a broader shift in which injury has been disentangled from questions of motive or moral causation in public health discourse (Green 1997, 1999). POM in particular is structured around a mandate to restore order, prioritising the protection of security forces themselves, rather than the wellbeing of those protesting (Brayford‐Harris et al. 2023). The medical consequences of police violence, when acknowledged, are treated as downstream events to be managed: the *ideal* situation is the absence of protest altogether.

This way of understanding both medicine and protest is structurally blind to the emergent forms of care generated during uprisings, especially when aligned with the aims of protest and openly confront state violence. Rather than a technical omission, this reflects what I term a security‐based medical approach to protest, where care is only legitimate insofar as it supports the restoration of order, not when it challenges sources of harm. In what follows, I contrast this paradigm with grassroots practices that reverse its assumptions. But before that, it is important to consider what becomes of medical neutrality, historically understood as a normative barrier between medical action and police and/or military forces.

### 1.2 Neutrality Under Strain

Neutrality has long underpinned medicine's self‐image as a profession. Rooted in Parsonian accounts of functional autonomy, neutrality is treated as an ethical imperative that legitimates professional authority (Freidson 1988; Parsons 1951). In this framing, at the macro level, the physician is insulated from the political stakes of the setting in which care is delivered. At the micro level, in relation to individual patients, they should respond to needs irrespective of the social or moral standing of the patient, what Parsons (1951) called ‘affective neutrality’. The performance of neutrality, inevitably, functions in part to elide what Beagan describes as biomedicine's role “in the historical oppression of all of those subordinated social groups defined as Other”  (2000, 1261).

Despite critiques of neutrality as ideological—concealing the social and political operations of biomedicine—it persists as a powerful normative force, evident in the fact that breaches require justification (Voutilainen et al. 2010). Where neutrality has been explored sociologically, it has largely been in contexts where autonomy is in question because professionals are employed in carceral or police institutions, that is, where there is an implicit alignment with the state rather than the patient (Gorga 2025). Such accounts underline the continued expectation that healthcare professionals remain neutral actors, oriented toward health and disease as apolitical states. Even in studies examining how professionalism has been reconfigured, neutrality remained a relatively underexamined, tacitly accepted trait—closely tied to medicine's public claims to objectivity and trustworthiness (Borgstrom et al. 2010; Elston 2009; Jones and Green 2006; Starr 2019).

In humanitarian settings, neutrality has been codified in international law and professional ethics. It is enshrined in the Geneva Conventions and reiterated by the World Medical Association, requiring medical personnel to treat the wounded impartially and prohibiting states from targeting or obstructing medical care (Yurdakul 2020). These principles remain deeply ingrained in public expectations, even in conflict zones where loyalties are often divided (Keshet and Popper‐Giveon 2017; List 2008). Yet this protective frame often assumes interstate conflict and mutual interest between warring parties in upholding medical neutrality, an assumption that breaks down in asymmetric, intrastate conflicts, where one party (the state) holds the monopoly of violence (Salunke 2020).

What becomes of neutrality, understood both as an ethical commitment and as an expectation of protection, as well as a key plank that legitimates claims for the authority and professionalism of healthcare provision, when the state itself, long seen as the shelter for professional development and autonomy, becomes the source of harm? And how do those on the ground—medical students, firefighters, nurses, rescue workers and civilian volunteers—navigate this rupture, when offering care becomes grounds for repression and the assumption of differential treatment of healthcare providers by the state collapses?

### 1.3 Protest and Health: Against the State

In contrast to security‐based approaches, recent scholarship has examined emergent forms of care practiced outside formal health systems during protest. Historical accounts show that medicine has long accompanied protest movements. In the United States, for example, Anderson and Cooper (2013) describe how doctors, nurses and medical students provided structured medical support during civil rights, anti‐war and Occupy protests—often through medical tents, accreditation systems and coordinated care. These and other examples (Rogers 2022) demonstrate commitments to solidarity with oppressed groups, but typically involved formal actors and professionalised operations. The cases examined in this paper, by contrast, reveal a distinct rationality, born from street‐level improvisation and distrust of institutions.

Like other such events, large‐scale protests turn familiar streets into contested spaces, disrupt circulation and overwhelm formal healthcare infrastructure, creating the need for grassroots care practices. Examples of this phenomenon include the Maidan Revolution in Ukraine in 2014, Chile's Estallido Social, large‐scale protests in Ecuador and Colombia between 2019 and 2021, the Hong Kong protests from 2019 to 2020 and the wave of anti‐racist protests across the United States in 2020. These events often escalate rapidly and involve violent confrontations, with police brutality becoming a central object of protest (Reynolds‐Stenson 2018).

Roborgh (2018), in her study of the Egyptian uprising in Cairo (2011–2013), describes the emergence of the *mīdānī* (square) doctor—a figure named after the *mīdān*, or public square, that became the epicentre of the protests. These doctors were not humanitarian outsiders, but locals whose authority stemmed from their presence in the square itself. Their dual identity as professionals and citizens and their continuous presence in the *mīdān*, blurred the boundary between care provision and protest participation. Similarly, Can (2016) and Aciksoz (2016) describe how volunteer physicians in Türkiye's 2013 Gezi Park protests became both healers and witnesses. Providing care in the midst of the protests exposed them to criminalisation, surveillance and physical violence by authorities. Their efforts to document injury and resist misinformation transformed the clinical act into a political intervention. Together, these cases reveal practices and motives behind healthcare action that differ from security‐based medical approaches to protest, practices developed by those who occupy a similar position vis‐à‐vis the state as the protesters themselves. By foregrounding this contrast, I explore what happens when healthcare emerges not from state planning, but from within the protest itself, rooted in the motives and conditions that call it into being. This is, I will argue, a fundamentally different way of conceptualising medical care in conflictual civic space, a ‘different position’ that re‐orients the place and nature of neutrality.

## 2 Methods

To develop the concept of protest medicine and explore what it might mean for understanding the relations of healthcare, the state and professional neutrality, I draw on in‐depth interviews with 21 people who voluntarily provided on‐site medical care to victims of police repression during the Estallido Social. The sample included medical, nursing and psychology students and professionals, as well as volunteers with backgrounds in engineering, firefighting and emergency response. Participants brought varying levels of prior training, ranging from formal clinical or tactical experience to informal, on‐the‐ground learning.

Participants were recruited through a combination of social media groups and indirect personal contacts, using snowball sampling, with the aim of capturing a diversity of entry points into different brigades operating in Santiago. Interviews were conducted between 2020 and 2023, either in person—during field visits to Santiago and key protest sites—or via videoconference platforms. All interviews were conducted retrospectively and invited participants to reflect on their experiences during the protests. Interview guides were semi‐structured and adapted over time, allowing questions to be tailored to participants' roles (e.g., clinical care, coordination, logistics) whilst maintaining a shared focus on experiences of care, risk and ethical decision‐making.

The analysis focused on exploring how protest was approached, understood and navigated by these participants and how their concrete practices and values were shaped by the emotional and political dynamics of protest.

All interviews were conducted and analysed in Spanish. Quotations presented in this paper were translated by the author, preserving tone and meaning. Interviews were transcribed and analysed thematically using Atlas.TI. The analysis combined deductive and inductive strategies: initial codes were guided by the project's interest in the actions and experiences of medical brigades during the protest events. Following Aronson's (1995) pragmatic approach to thematic analysis, transcripts were reviewed for recurring ideas and related codes were grouped into broader topics. These were not based on frequency but on conceptual coherence, linking fragments of experience into interpretive themes that reflected how participants made sense of their actions and ethical stances.

To structure the analysis, the material was organised into three descriptive domains: (1) the emergence and coordination of medical brigades; (2) experiences of police hostility and its influence on brigade action; and (3) the emotional, ethical and political dimensions of care. These domains served as an intermediate analytical step, helping the transition from coding to writing. In the findings section, these were reworked into four analytically distinct but overlapping sections that foreground the organisational, experiential and ethical‐political dimensions of protest medicine.

All participants gave informed consent to participate in the study. The project received ethical approval from the Research Ethics, Governance and Compliance department at the University of Exeter. Interviews were conducted under conditions of anonymity; pseudonyms are used throughout and participants were informed of their right to withdraw. Audio recordings were securely stored and deleted after transcription and anonymised transcripts were archived in accordance with institutional data protection protocols. In the findings, participants are identified by pseudonym and, where relevant, by their professional or organisational role, in order to situate their testimonies within the analysis.

Some organisations have also been deidentified, particularly where naming them would risk identifying individual members. The only inclusion criterion was direct involvement in medical or psychological care during protests. The aim was to capture varied perspectives and modes of participation across different brigades and phases of the uprising.

The following sections draw on participants' testimonies to offer a grounded and intimate account of how care was mobilised in response to the extraordinary violence and emotional intensity of the events. What emerges is a complex picture of tactical coordination, emotional intensity and ethical reflection. The accounts span the period from October 2019 to early 2020, tracing the rapid emergence of brigades in the first weeks of the uprising, their consolidation during peak mobilisation and their gradual decline with the onset of COVID‐19.

### 2.1 From Chaos to Care: The Birth and Co‐Ordination of Medical Brigades

When the protests started, some groups with prior emergency response experience mobilised rapidly, drawing on pre‐existing networks‐although not always under their original organisational identities. Sebastián Ríos, a nurse and firefighter, for instance, was part of a well‐established NGO with a background in disaster relief. When the scale of injuries became apparent, he proposed mobilising the group:

I am a firefighter. I also belong to an NGO that has already responded to various emergencies in the country. (…) When all this social upheaval started, there began to be many injured people. A colleague told us there were many wounded with no one to care for them. He asked the people of the NGO and to the fellow firefighters, saying, “You know what, there are many injured people. We should take this as another emergency and we should provide aid”.

But, he reported, the NGO had refused, citing reputational risk due to the political nature of the protests. This hesitation led Ríos to act independently, co‐founding *Equipos de Rescate Voluntario* (Voluntary Rescue Teams, ERV), which quickly became one of the most visible and effective collectives of the period. Indeed, only one existing NGO, *Red de Salud en Crisis* (Health in Crisis Network, RSC), joined the protests under its formal identity. Framing the uprising as a public health emergency, RSC maintained a humanitarian, professional stance, whilst also supporting the organisation of care on the ground through training and coordination. As Marcelo Torres, a nurse and academic involved in the group, explained:

We, professors, were contacted by RSC students who said, 'This is a catastrophe from a health perspective; we have to do something.' We prepared equipment and set up a place where injured people could come to us, instead of wandering around looking for injured individuals.

Aside from RSC, most NGOs avoided involvement, unwilling to blur professional and political boundaries. This reluctance reflects the enduring power of medical neutrality as an ethical principle and a professional safeguard. Yet, this boundary is not fixed. Medical neutrality is often challenged, eroded, or strategically invoked depending on context (Benton and Atshan 2016; Bricknell 2024; Hamdy and Bayoumi 2015). In this case, most NGOs viewed involvement in the protests as a threat to their professional legitimacy, fearing that association with politically charged events might compromise the apolitical image on which their social authority depends.

In this vacuum, a new form of grassroots healthcare provision emerged: the *brigada médica* (medical brigade) or *brigada de salud* (health brigade). In Latin America, particularly in Cuba, the term often refers to formal international medical missions providing clinical, educational and system‐strengthening support in disaster settings (Céspedes 2024; López et al. 2014). In contrast, Estallido Social brigades were informal, self‐organised collectives, formed in protest squares, initially without institutional backing or central coordination.

Although some participants brought experience from fields such as emergency response or healthcare, the brigades themselves were assembled in real time. As Rafael Contreras, an expert in rescue operations who later trained some of the brigades, recalled: “Of all the people who were helping, maybe 1% had any real training. The rest were ordinary people—neighbours, bystanders, whoever happened to be there.” Similarly, medical student Diego Herrera described the early response as improvised and fluid: “Everything started spontaneously. It wasn't something planned; it was built along the way. At the beginning it was very rudimentary—everything was chaotic”.

As the protests unfolded and the number of injuries increased, many groups began to organise more systematically, with defined roles and protocols. This shift is evident in the account of Víctor Ríos from ERV. Over time, he explained, the group developed regular routines. These included forms of evaluation and training, the rudiments of a developing clinical autonomy for the brigades:

We conducted daily meetings after each action to analyse what went well, what we needed to improve and where we were failing. (…) In the mornings we provided first aid training, teaching volunteers how to control bleeding or bandage wounds and in the afternoons, we went out to attend to the injured.

This evolving organisation reflected a process of functional professionalisation (Salvatore et al. 2018), as groups began to adopt practices traditionally associated with formal healthcare. Coordination became critical to manage increasingly complex injuries, ensure volunteer safety and logistical efficiency. Yet this emerging professionalism was never static. The volatility of protest demanded constant responsiveness. Adaptation happened continuously, sometimes through collective restructuring at the brigade level or individual improvisation in the field. As Nicolás Paredes, a psychiatrist, recalled, his role transformed in real time: “I went to support psychiatric needs initially, but I ended up working more generally as a doctor, treating people with complex injuries, alongside medical students”.

As more brigades formed, they differentiated their roles and cooperated with each other. Larger bodies such as The Federación de Estudiantes de la Universidad de Chile (University of Chile Student Federation—FECH) and the Colegio Médico (Chilean Medical Association, COLMED) contributed to training, coordination and the provision of infrastructure. FECH, for instance, turned its own facilities into a dedicated care and support centre, whereas COLMED facilitated inter‐brigade meetings and helped standardise practices across groups. Rodrigo Salas (ERV) detailed the process and the role of his brigade in this network:

At the facilities of the COLMED, we held a meeting with all the brigades (…) to achieve standardisation, get to know each other and decide what we were going to do. (…) Our own task as a brigade was extraction: approaching, retrieving, stabilising [injured people] as much as possible and then extracting [them] to a safe point for further care.

Brigade members also recalled the efforts to train new members. As Marcelo Torres highlighted, this structured training became mandatory to ensure readiness and safety, especially for students: “In RSC we developed a training plan. Any student arriving at the post had to have completed the plan. They had done courses on basic CPR, life management, mobilization and transfer. Not just any student could join”.

Training included essential operational skills. Rafael Contreras led sessions on emergency transfers and on‐the‐ground decision‐making. As he explained, “Transferring someone in the street with an active shooter or massive crowd isn't the same as in controlled situations. In emergencies like these, there's no time for politeness—you must be direct and logical.” Although traditional emergency medicine discourages moving victims with potential injuries, Contreras emphasised that “during protests, the logic changes entirely; you must evacuate victims immediately because waiting might lead to death”.

Inter‐brigade coordination also involved the need for symbols for reciprocal recognition, visible identifiers of brigade status and tactical communication systems. Andrés Molina described how these efforts were modelled on international norms:

We began setting standards immediately. I said, 'Look everyone, we have to identify ourselves.' Because according to the laws of war, all medical personnel must be clearly identified. From the next day, everyone wore yellow jackets, helmets with a red cross and identification tags.

As brigades expanded, care was organised through a distributed network of fixed and mobile points. Participants described multiple sites operating simultaneously, ranging from small first aid posts to more complex spaces resembling improvised field hospitals. Locations included university buildings, cultural centres, small commercial premises and residential passages repurposed as treatment and supply hubs. Some sites, such as those coordinated through FECH or spaces like Teatro del Puente, developed internal triage systems, treatment areas and basic resuscitation capacity, whereas others functioned as extraction or decontamination points closer to the front‐line. These sites were not insulated from violence: several were exposed to tear gas, police incursions, or surveillance, requiring constant relocation. As noted by Diego Herrera, medical student involved in brigade coordination, “the map changed with every protest”.

Yet these symbols and infrastructures developed in response to violence offered little protection. The next section explores how medical brigades, despite their initial efforts to adhere to principles of neutrality, became direct targets of state violence.

### 2.2 Medical Brigades as Targets of State Violence

Although participants in Chile's brigades came from diverse backgrounds, including nursing students, firefighters, rescue volunteers, psychologists, neighbours, medical neutrality still shaped how many approached their role. Yet as the Estallido unfolded, this expectation—deeply embedded in both professional medicine and humanitarian law—was repeatedly undermined.

Across the interviews, a recurring progression was described: first, the shock at the scale and indiscriminate nature of the violence and then the realisation—more devastating still—that even those offering care were not exempt. “We thought they would at least respect us,” one participant reflected. “But there was no distinction. We were just another target.” Many volunteers assumed that the use of medical symbols would grant them immunity, as it might in disaster zones or wartime. That belief was quickly shattered. Police not only failed to recognise their neutrality, they actively targeted medics. As Sebastián Ríos put it, “The police respected nothing—not even the Red Cross. International norms were never respected in this conflict”.

Although some brigades were stationed behind the lines, many remained close to the “hot zone”, exposed to the same dangers as the protesters they assisted. Camila Fuentes, a nursing student, described how providing care often meant becoming a target: “We were doing first aid and a *carabinero* [police officer] saw me and shouted, ‘Are you doing first aid? Suck it!’ and fired the tear gas canister directly at me”.

Paula Navarro, a nursing student during the protest events, recalled one incident that marked her especially: “We were doing CPR in the middle of the street and even though we were waving white cloths, they still tear‐gassed us… It was incredibly distressing.” Medics were not only beaten and tear‐gassed but, in some cases, sexually harassed. During one dispersal, Camila was groped by police: “They pushed me, grabbed my chest and my backside. I didn't process it at the time, I was focused on others. But later I realised what had happened.” Reflecting on the indiscriminate nature of the repression, she said: “There was no logic to the violence. It wasn't about stopping people who were doing harm. Anyone could be hit.” Diego described the fear that permeated their work: “We were afraid they'd come after us for helping people. But we couldn't stop. Our ethical commitment to care was stronger than our fear”.

Realising they were being targeted reshaped how brigades approached safety. Ríos recalled: “We all equipped ourselves—helmets, goggles, knee pads, elbow pads, tactical vests, gloves, respirator masks. By the end, we were using full‐face 3M masks with filters.” But protective gear only addressed part of the risk. Medical posts attacked, mobile units harassed and medical equipment seized. “They tried to run us down with the water cannon,” Sebastián said. “Forty of us had to form a shield line with whatever we had”.

Over time, brigades developed internal security protocols to protect themselves. These involved formalising entry for brigade members, tracking shifts and adopting QR systems to document movement and supplies. The FECH headquarters became a logistical hub, but the risk of infiltration and the political visibility of care, meant no space ever felt fully safe. As the repression intensified, some volunteers stepped back or sought safer posts. Others, like Camila, chose to remain as close as possible to the front‐line. “We didn't want to be in the safe zone,” she said. “We wanted to be where it was happening. Not in the first line, but right next to it, to get there fast, to be where we were needed”.

Participants described a strained relationship with the formal health system.

Hospitals near protest zones, particularly Posta Central, were rapidly saturated: “we were overwhelming the emergency services… there were many, many, many” (Sebastián Ríos).

Specialised services, such as the ocular trauma unit at Hospital del Salvador, also reached collapse. Although hospitals remained essential for severe cases, access was often delayed or avoided. Some brigades established informal coordination with ambulance teams (SAMU) and in some cases personnel joined as volunteers. Despite this, distrust toward institutional care persisted, shaped by fears of surveillance, police presence, or loss of control over patients. As a result, hospitals functioned simultaneously as sites of necessary care and as spaces approached with caution.

### 2.3 Care Under Pressure: Emotion, Fatigue and Solidarity

The emotional experience of participating in the medical response was as intense and disorienting as the physical violence. Nicolás Paredes, a psychiatrist and one of the mental health professionals coordinating field care, offered a striking metaphor to describe this collective state of mind: “Our vision narrowed, we entered a crepuscular state, our awareness was reduced to a single thing, one circle, one environment.” Drawing from psychiatric language, the idea of a crepuscular or “twilight” state speaks to a state of altered consciousness where ordinary coordinates of time, self and action blur. Perception becomes saturated, lucidity persists yet feels dreamlike and urgency overrides deliberation. Protest, for many, became a closed, totalising emotional world. Expanding on this, Nicolás explained how he would urge injured protesters to go home and protect themselves: “You could get killed, something could happen to you and you've got a whole life outside of this.” Still, many refused. “You're limping with a bullet,” he would tell them, “if they chase you, you won't be able to run.” Protesters weren't willing to step away, though, even when wounded. Reflecting on his participation, he admitted he was no different: “It happened to all of us.” Immersed in the atmosphere of protest, he too lost perspective. “I was absorbed in it for months, until I started to feel really bad.” He recalled his rapid weight loss, emotional exhaustion and blurred personal limits: “With the adrenaline, you'd skip meals, lose your appetite and just keep going, just keep showing up. We were all the way in”.

Others, like Paula Navarro, sustained themselves through collective support and recognition. A nursing student at the time, she recalled how trauma accumulated quietly, day by day. “We were going almost every day… I'd get home crying or feeling overwhelmed from the things I'd seen.” She described how the weight of everyday trauma built up gradually until it became overwhelming. “There was a serious kind of emotional exhaustion,” she recalled. “More than not knowing what to do, it got to a point where we'd been going almost every day for a month—I'd come home crying or filled with anguish from the things I had seen.” The daily toll was physical too: “My legs hurt, my arms, my back.” Still, her memories are also marked by moments of deep solidarity:

There were times when people clapped for us, when they helped us—They’d make way when we came through with someone injured, or they’d shout and wave to let us know where someone needed help. There was this feeling of citizen collaboration and it was beautiful.

Despite the pain and despite the fatigue and fear, she continued for as long as she could: “There was something very powerful about being there, about feeling like we were making history”.

Solidarity extended not only across protest lines but within the brigades themselves. Trust, humour and mutual care became essential survival strategies. As Nicolás put it, “We became something like a family.” They shared fears, food, jokes, advice and conversation. Some stayed for months, until the pandemic put an end to the protests, in March 2020. Others, like Nicolás, left when the burden became too heavy. “Looking back, it was incredibly intense. I'm not sure I'd do it again. But it was a defining moment in my life.” For many, the experience transformed how they understood medicine, care and their own place in the world.

### 2.4 Professional Ethics Under Fire: The Rise of Political Care

This final section builds on the previous domains to explore how participants' ethical orientations were challenged and reshaped as neutrality became untenable. The medical brigades' experience during the Estallido Social exposed the limits of traditional notions of neutrality as a core value of professionalism. Neutrality was first denied and then disavowed, as brigades reoriented their ethic of care. Many volunteers began with a clear ethical stance that echoed that of humanitarian medicine: to treat the injured, whoever they were. Paula Navarro recalled, “I didn't care who it was—whether they were frontliners, someone with a family, a police officer. If they were hurt, I wanted to help.” Diego Herrera echoed this view: “For me, giving aid wasn't political—it was ethical. I treated whoever needed it.” Rodrigo Salas even recalled treating injured police officers in the early days: “We tried to apply the principle of humanitarian aid. We treated four of them—but after that, they wouldn't let us get near them again. It was showing too much that we weren't the monsters they said we were”.

As the repression intensified, neutrality became increasingly untenable for many brigade members. Sebastián Ríos described the shift: “We came out strictly to help the wounded—but then we started getting attacked by the police. They'd shout things at us even when we were working.” Many came to realise that their visible role as healthcare providers did not shield them—it marked them: “We witnessed serious human rights violations and many of us had to testify. The police identified us—and that changed everything. They beat us. They assaulted our colleagues”.

This rejection of medical neutrality by the state forced a reckoning. Some brigade members held onto impartiality as a moral ideal, but began to question its practical relevance. Although many continued to affirm that care should be provided regardless of who was injured, their experiences strained this position. Others described a tension between treating everyone and protecting those targeted by police violence. As Camila Fuentes put it, “It wasn't just treating wounds—it was protecting people from the police.” In this sense, impartiality did not disappear but was reworked under conditions of asymmetric harm and repression.

These experiences echo what Salunke (2020), building on Redfield (2013) has observed in other settings: that medical neutrality is a vestige of interstate war that no longer functions in intrastate asymmetric conflicts. The conditions that once supported medical neutrality no longer hold in many contemporary protest and repression scenarios. As she writes, “As a product of the state, doctors are investments that the state chooses to call upon. And, because the state is in the position of power within the frame of the state‐civilian conflict, they will inevitably put pressure on the medical community to serve their interests” (p6). In these conditions, treating injured protesters becomes a form of defiance and is punished accordingly.

For some, the experience sharpened the political implications of their professional identity.

Psychiatrist Nicolás Paredes reflected: “My stance is that I'm a doctor first, no matter my political views. If someone needs care and I can provide it, I will—whoever they are.” Yet in the same breath, he acknowledged how his experiences had blurred that boundary: “Being a doctor is political. I couldn't stay neutral in the face of what was happening.” His words reflect the friction between a professional ideal of neutrality and the demands of a context where neutrality itself is compromised.

Yet it was not only their immediate clinical labour that became politicised. As Redfield (2013) argues in his study of MSF, *témoignage* [testimony] has long been a central form of medical engagement in crisis settings. Brigades in Chile embraced a similar ethic. They collected injury data, documented cases, archived evidence and shared testimonies that would otherwise be buried by institutional silence. Sebastián Ríos said: “We kept a record of all the wounded—more than ten thousand.” Rodrigo Salas, from the same brigade, described sending water samples from police water cannons for international toxicology testing after chemical burns appeared in protesters. These acts were not mere bureaucratic exercises—they were political interventions. As with MSF's témoignage, these actions constituted an “affirmation of facts” through professional expertise, challenging state impunity from within the very domain the state sought to suppress (Redfield 2013).

Salunke (2020) similarly emphasises how professional authority over the body grants medical workers the ability to resist and subvert oppressive narratives once neutrality is no longer viable. Neutrality can become active neutrality on the side of victims. For Chile's brigades, this meant transforming their roles—not by abandoning professionalism, but by weaponising it: using clinical expertise and professional authority to treat, document and bear witness to state violence. As such, brigade members occupied the protest differently to other groups: not as police enforcing order, nor as journalists documenting events and not exactly as protesters demanding change, but as embedded caregivers responding to harm as it unfolded. Their perspective was shaped by the necessity of making sense of pain and injury, from within the protest itself. They saw what others could not: the patterns of state violence, the rhythms of repression, the costs borne by bodies including their own.

The motivations that brought people into the brigades were multiple and sometimes contradictory: humanitarian concern, political conviction, professional ethics, rage, fear. Their backgrounds and trajectories diverged widely. Despite this diversity, a common thread emerged: the act of providing care during protest became a moral and political response to immediate harm, shaped by proximity to violence and by the failure of institutional protections.

## 3 Discussion

This paper has traced how protest medicine, as it emerged during Chile's Estallido Social, was not simply an improvised response to crisis but a situated reconfiguration of medical ethics, practice and meaning. The brigades' work challenged dominant assumptions about what medicine is and what it should do when the state becomes a source of harm. Their clinical knowledge and ethical commitments did not vanish but were reoriented in the face of violence and the failure of institutional protection. The entanglement between professionalism and resistance did not simply strain the idea of medical neutrality; it redefined what it meant to care when the state was the source of harm.

In this final section, I revisit the broader stakes of these findings—examining how protest medicine unsettles a security‐based approach to medicine during protests, reframes medical neutrality as both a moral claim and a political position and points toward a reimagined professional ethics rooted in solidarity and confrontation rather than detachment. What follows develops these arguments through three interconnected lenses: the reinvention of medical practice under repression, the collapse and strategic retooling of neutrality and the broader implications for how we understand medicine's social contract under conditions of state violence.

### 3.1 Inventing Care in a Hostile Terrain

If medical brigades became witnesses and agents of solidarity, it was because the protest terrain made conventional care unworkable. During the Estallido Social, the assumptions underpinning standard emergency response—open access, predictable injury, provider neutrality—did not hold. Protesters avoided hospitals out of fear of surveillance or detention. Ambulances were blocked, attacked, or mistrusted. Medical posts were targeted. The very foundations of medical neutrality, central to security‐based frameworks like POM and others (Brayford‐Harris et al. 2023), collapsed in real time and, in response, brigades did not merely adapt existing protocols; they invented new ones. They created mobile teams, retooled everyday objects as medical equipment and transformed cultural centres and university spaces into makeshift clinics. Protocols were established to accommodate hostilities: treating trauma whilst under fire, evacuating patients through gas clouds, protecting them from police attacks.

This reconfiguration was forced by the situation. First, protest presence and confrontation with the police made transport (including ambulances) impossible for the duration of the protests. Second, widespread distrust in institutions, fuelled by credible fears of surveillance and retaliation (Amnesty International 2020; Benedicto and Garcia 2022; Instituto Nacional de Derechos H and umanos 2019), undermined consistent reliance on the state's medical infrastructure. Third, a civic ethic of self‐support, grounded in Chile's long‐standing traditions of autonomous collective solidarity in the face of natural disasters (Caro Zúñiga et al. 2021), gave ethical coherence to care under fire. Although such solidaristic repertoires are not unique to Chile (Albris 2025; Bartolucci and Magni 2017), their specific historical and cultural articulation helped sustain collective action in this context and may shape how similar forms of protest medicine emerge elsewhere. Together, these conditions constituted the terrain on which protest medicine was forged.

This care did not arise in the vacuum left by the state; it emerged in *defiance* of it. It was a refusal to separate medical action from political responsibility, to accept the framing of protest as mere disorder, or care as inherently apolitical. In this sense, protest medicine foregrounds a form of healthcare rooted in proximity, solidarity and resistance rather than neutrality, regulation and order.

### 3.2 Care Beyond the State: Rethinking Medicine Through Protest

Across testimonies and settings, protest medicine during Chile's *Estallido Social* was a refusal to abandon care when institutions faltered and a reinvention of what care could mean under fire. What is distinctive about this form of care is that it takes shape within the field of medicine—a domain conventionally anchored in neutrality, legality and state legitimacy—and turns those premises inside out. Emerging in response to institutional abandonment and active repression, protest medicine operated outside the protections typically afforded to medical personnel and often in defiance of legal and professional norms (Macauley 2005). In doing so, it unsettled a longstanding vision of the relationship between medicine and society, often described as a ‘social contract’—a framework through which society, mediated by the state, grants the profession authority, autonomy and status in exchange for expertise, ethical commitment and service to the public good (Bhugra 2024; Cruess and Cruess 2004; Dutta et al. 2025). In this case, protest medicine operated outside the limits of this arrangement, grounding care not in institutional legitimacy, but in solidarity, moral urgency and resistance (Ellaway and Wyatt 2022). Rather than a relationship mediated by the state, the social contract is reoriented toward those subjected to harm: professional authority is no longer secured through alignment with state structures, but through direct accountability and proximity to suffering.

In this sense, what developed in the protest squares was a shadow health system that combined tactical improvisation, embodied expertise, emotional labour and political consciousness. It redirected long‐standing traditions of Chilean mutual aid toward surviving state violence. The refusal to delegate care to institutions seen as complicit created new spaces, solidarities and ways of imagining medical action. Taken together, these insights make it possible to articulate a synthetic definition of *protest medicine* as a distinct and evolving form of medical practice.

What follows is a provisional framework aimed at capturing the key features of this emergent form of care whilst remaining attentive to its plural and unfinished character: Protest medicine is the voluntary, improvised and politically entangled provision of healthcare that arises within episodes of collective unrest and state violence. It emerges when official health systems are perceived as inadequate, unreliable, or hostile and when the moral and professional obligation to treat overrides legal or institutional boundaries. It is practised by an assemblage of actors—health professionals, students, firefighters, rescue workers and lay volunteers—and is sustained by solidarity rather than hierarchy and by proximity to harm rather than institutional distance.

Whether in Cairo's *mīdān* (Roborgh 2018), Istanbul's Gezi Park (Aciksoz 2016; Can 2016), Kyiv's Euromaidan (Stepurko et al. 2014) or Santiago's Plaza Dignidad, protest medicine departs from a security‐based framing of protest as disorder and care as a neutral technical response (as in MGM or POM). Instead, it treats harm as political, care as embodied solidarity and medicine itself as aligned with resistance to oppression. Rather than upholding or prioritising its relationship with the state, protest medicine imagines—and enacts—alternative infrastructures of care. It transforms clinical knowledge into political practice, builds new solidarities under fire and expands what medicine can mean when it breaks from the state's moral and logistical constraints. It is, at once, a field intervention, an ethical stance and a reimagining of medicine's social contract.

This article offers one possible theorisation of protest medicine, rooted in the empirical specificity of Chile's Estallido Social. However, the category is neither fixed nor exhaustive. Future research might explore contrasts with other figures—such as indigenous medics during anti‐colonial uprisings, military doctors in conflict zones, or activist clinicians in contexts of state violence. These perspectives could illuminate further tensions between medical ethics, professional identity and political resistance. What is at stake in these formulations is not only how care is practised, but how medicine itself is imagined—as a domain either bound to state legitimacy or open to rearticulation through popular struggle.

## 4 Conclusion

This paper has examined the emergence and practice of protest medicine during Chile's Estallido Social, showing how informal medical brigades reconfigured care in response to sustained state violence. Based on the testimonies of those who provided treatment, protection and documentation under fire, the analysis reveals how medical practice was transformed by the spatial, emotional and political conditions of protest.

Far from acting as substitutes for absent institutions, brigades developed a form of care that refused the institutional logics of neutrality and detachment. Protest medicine in Chile was not only improvised or reactive: it was a form of embodied dissent. It exposed the ambivalent role of formal health systems, including their limitations, entanglements with state power, and, at times, forms of complicity, whilst rendering harm visible through situated forms of witnessing, solidarity and ethical urgency.

By tracing how care was enacted beyond and against state control, this study contributes to broader efforts to rethink the role of medicine when state and police legitimacy is in crisis. Protest medicine, as theorised here, is a distinct form of medical action: one that arises from within protest, responds to repression and is sustained by collective risk, solidarity and refusal. It not only reimagines how care is delivered, it expands what care can mean in times of political rupture.

## Author Contributions

**Cristian Montenegro:** conceptualization, investigation, funding acquisition, writing original draft, methodology, validation, visualization, writing – review and editing, software, formal analysis, project administration, data curation, supervision, resources.

## Conflicts of Interest

The author declares no conflicts of interest.

## Data Availability

The data that support the findings of this study are available on request from the corresponding author. The data are not publicly available due to privacy or ethical restrictions.
